# Dietary Restriction Extends Lifespan in Wild-Derived Populations of *Drosophila melanogaster*

**DOI:** 10.1371/journal.pone.0074681

**Published:** 2013-09-10

**Authors:** Athanasios Metaxakis, Linda Partridge

**Affiliations:** 1 Max Planck Institute for Biology of Ageing, Cologne, Germany; 2 Institute of Healthy Ageing and Department of Genetics, Evolution and Environment, University College London, London, United Kingdom; University of Lausanne, Switzerland

## Abstract

Dietary restriction (DR) can result in lifespan-extension and improved function and health during ageing. Although the impact of DR on lifespan and health has been established in a variety of organisms, most DR experiments are carried out on laboratory strains that have often undergone adaptation to laboratory conditions. The effect of DR on animals recently derived from wild populations is rarely assessed. We measured the DR response of four populations of *Drosophila melanogaster* within two generations of collection from the wild. All populations responded to DR with an increase in lifespan and a decrease in female fecundity, similarly to a control, laboratory-adapted strain. These effects of DR are thus not a result of adaptation to laboratory conditions, and reflect the characteristics of natural populations.

## Introduction

Dietary restriction (DR), a reduction of nutrient intake without starvation or malnutrition, was first shown to lengthen life in rodents [1]. DR has since been shown to extend lifespan in diverse organisms, including yeast, nematodes, flies and rodents [2]–[7]. Importantly, DR also delays the onset of age-related functional decline and disease in rodents and primates [8]–[10].

Extension of lifespan by DR is a well established phenomenon, but much of the information has been derived from long-established laboratory strains, which can evolve rapidly in response to laboratory culture [11]. Although DR had a clear lifespan-extending effect in wild-derived worms [12], wild-derived rodents showed increased maximum, but not mean, lifespan under DR [13]. This finding raises the possibility that the anti-ageing effect of DR in some species could be an artifact of adaptation to laboratory culture. Furthermore, DR has been shown to decrease rather than increase mean longevity in several recombinant inbred mouse strains [14], indicating that genetic variation associated with inbreeding can be an important determinant of the effects of DR.

In budding yeast [15], worms [4] and *Drosophila* [6], the response of lifespan to DR is tent-shaped, initially increasing as food intake is reduced from a high level, and then declining with the onset of starvation. At least in *Drosophila*, the food intake level at which lifespan peaks can vary between different laboratory strains [16]. A wide range of food intakes must thus be sampled to characterise the response of a particular strain to DR. In studies of DR in mice, usually only two levels of food intake are used, and the range over which a response to DR occurs could therefore easily be missed.

*Drosophila* has proved a useful model organism for studies of the mechanisms of DR [17], [18]. Adaptation of the fly life history to the standard method of laboratory culture with discrete generations results in shortened lifespan [19], [11], although this can be prevented by continuous culture in population cages [11]. Flies cultured in cages show a consistent response to DR while, in contrast, several of the standard laboratory inbred strains show a more attenuated set of responses [20]. It is therefore important to establish the responses to DR of fly strains newly derived from nature.

To address this question, we captured flies from four different locations in Europe and examined the response of their second-generation descendants to DR. All four strains showed a typical tent-shaped response of lifespan to DR through yeast dilution, together with a decrease in fecundity, similar to the responses of a control, laboratory-adapted strain. We therefore conclude that these effects of DR in *Drosophila* are not an artifact of laboratory culture.

## Methods

Collections were made from four different geographic locations in Europe, in Germany, Netherlands, France and Greece (**Table 1**). No specific permits were required for the field collections and permission to collect samples on private land was provided by Gabi Hatman, Anna Metaxaki, Michael Daskalakis and Linda Partridge. The field collections did not involve endangered or protected species.

**Table 1:** *Drosophila melanogaster* wild-derived strains used in this study.

Strain	Origin
FRA	Compost heap in France (43° 45' 0"N/3° 26' 0"E).
GER	Garden in Cologne, Germany (50° 56' 0"N/6° 57' 0"E).
GRE	Wine cellar in Crete, Greece (35° 20' 0"N/25° 8' 0"E).
NETH	Garden in Eindhoven, Netherlands (51° 27' 0"N/5° 28' 0"E).

Wild-derived flies were reared in population cages [20] (flies collected in France) or in glass bottles (flies collected in Greece, Netherlands and Germany), and grand offspring of wild-derived flies were used in the DR experiments. To rear the experimental flies, eggs were collected and distributed into bottles of 1X SY food at standard low density [21]. After eclosion, adult flies were allowed to mate for 48h. On the third day of adulthood, mated female flies were anaesthetized with CO_2_, and set up at 10 per glass vial. For each food condition we measured the lifespan and fecundity of 100 female flies. We included for comparison a laboratory-adapted, *white* Dahomey strain [20], derived from flies collected in Dahomey (now Benin) in 1970 and maintained since in large population cages with overlapping generations on a 12 L : 12 D cycle at 25°C.

To implement DR, flies from each strain were maintained on Sugar/Yeast/Agar (SY) food [22] with varying concentrations of yeast. Dilution of yeast alone is sufficient to induce DR in flies, decreasing fecundity and first increasing then decreasing lifespan [22], [23]. Five concentrations of yeast (0.1, 0.5, 1.0, 1.5 and 2.0) were used, where 1 corresponds to 100 g/L brewers’ yeast (MP Biomedicals). This range of yeast concentrations has captured the DR responses of all fly strains so far tested [22]. Moreover, this strain of baker’s yeast has been shown to be optimal for fly culture and DR experiments [16].

To measure lifespan, flies were transferred to new vials three times per week at which time deaths were scored. Fecundity for the first month of flies’ life was estimated from egg-counts made five times for each strain, twice during the first week of adulthood and once in each of the three following weeks. The mean number of eggs laid/fly/day was calculated. Lifespan and fecundity data were analysed using ANOVA, with strain and food level as fixed main effects in Graph Pad Prism 5.03 software (Graph Pad Prism Software Inc.). Pairwise comparisons were performed with 2-tailed Student’s t-tests. Multiple comparisons among strains were performed with one-way ANOVA with Bonferroni's Multiple Comparison test. Regression analysis was performed in Graph Pad Prism 5.03 software (Graph Pad Prism Software Inc.). For linear contrasts analysis with Bonferroni's test we used SPSS Statistics for Windows, Version 17.0, Chicago: SPSS Inc. Survivorship data were analysed in Excel using the Log Rank test.

## Results

### Wild-derived flies showed a tent-shaped response to DR

**Figure 1:**
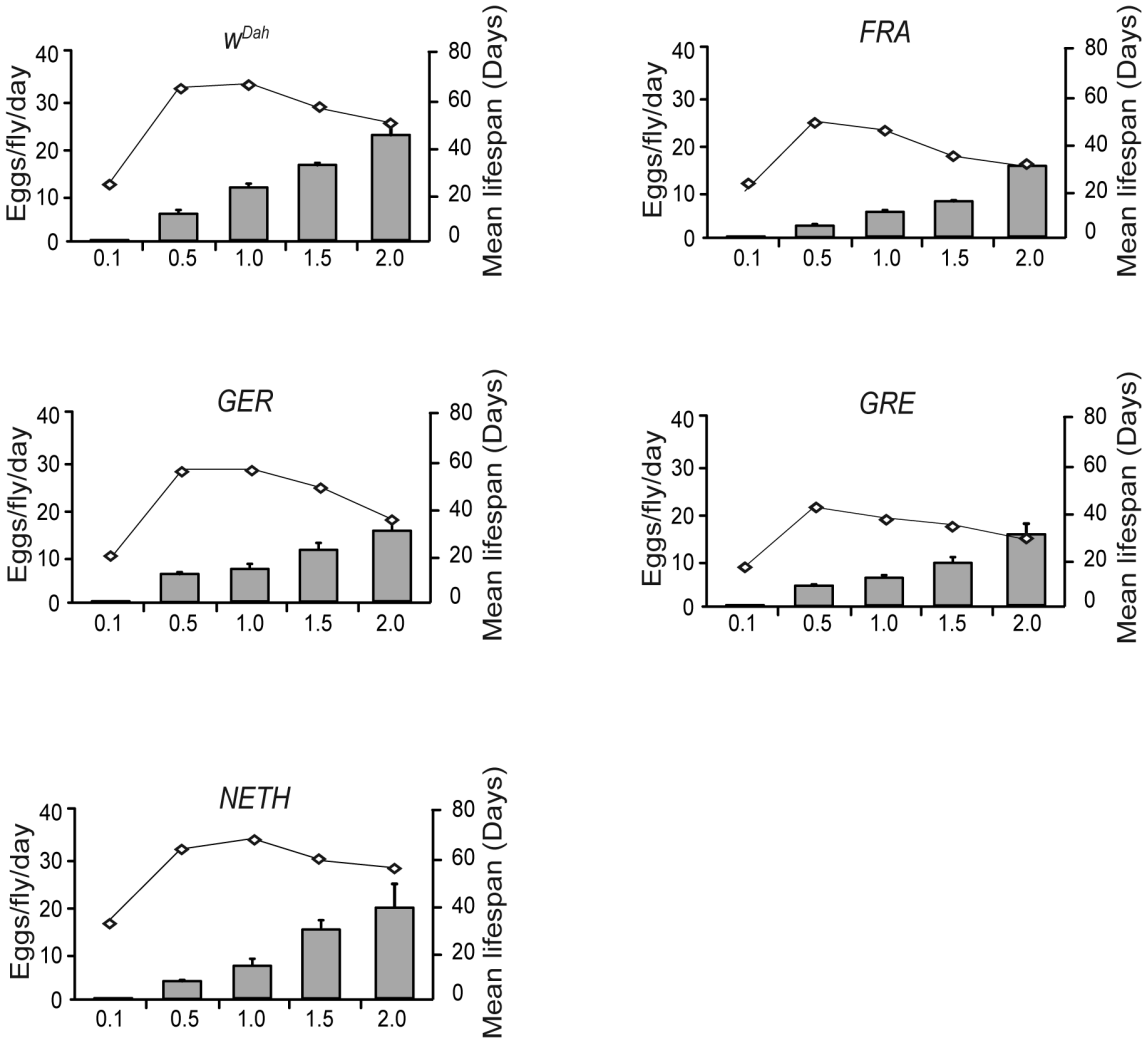
Lifespan and fecundity of wild–derived *Drosophila melanogaster* strains responded to DR. The mean lifespans of all wild-derived strains and the laboratory strain exhibited a tent-shaped response to DR with highest mean lifespan values at 0.5 or 1.0 yeast concentration. Female fecundity showed a monotonic increase with yeast concentration in all strains. Both mean lifespan and fecundity were significantly affected by food. Mean lifespan for *W^Dah^*: F = 94.21, R^2^ = 0.8933, *p*<1×10^−4^, for *FRA*: F = 35.42, R^2^ = 0.7589, *p*<1×10^−4^, for *GRE*: F = 81.23, R^2^ = 0.8784, *p*<1×10^−4^, for *GER*: F = 96.66, R^2^ = 0.8957, *p*<1×10^−4^, for *NETH*: F = 43.25, R^2^ = 0.7936, *p*<1×10^−4^, one-way ANOVA test. Fecundity for *W^Dah^*: F = 136.5, R^2^ = 0.9239, *p*<1×10^−4^, for *FRA*: F = 158.3, R^2^ = 0.9337, *p*<1×10^−4^, for *GRE*: F = 103.1, R^2^ = 0.9016, *p*<1×10^−4^, for *GER*: F = 107.6, R^2^ = 0.9054, *p*<1×10^−4^, for *NETH*: F = 123.5, R^2^ = 0.9165, *p*<1×10^−4^, one-way ANOVA test. Points: mean lifespan. Bars: estimate of mean number of eggs laid/fly/day ± standard error; connected points: mean lifespan in days (*n*  = 100). Data shown are from a single trial with second generation flies of the wild-derived strains.

**Figure 2:**
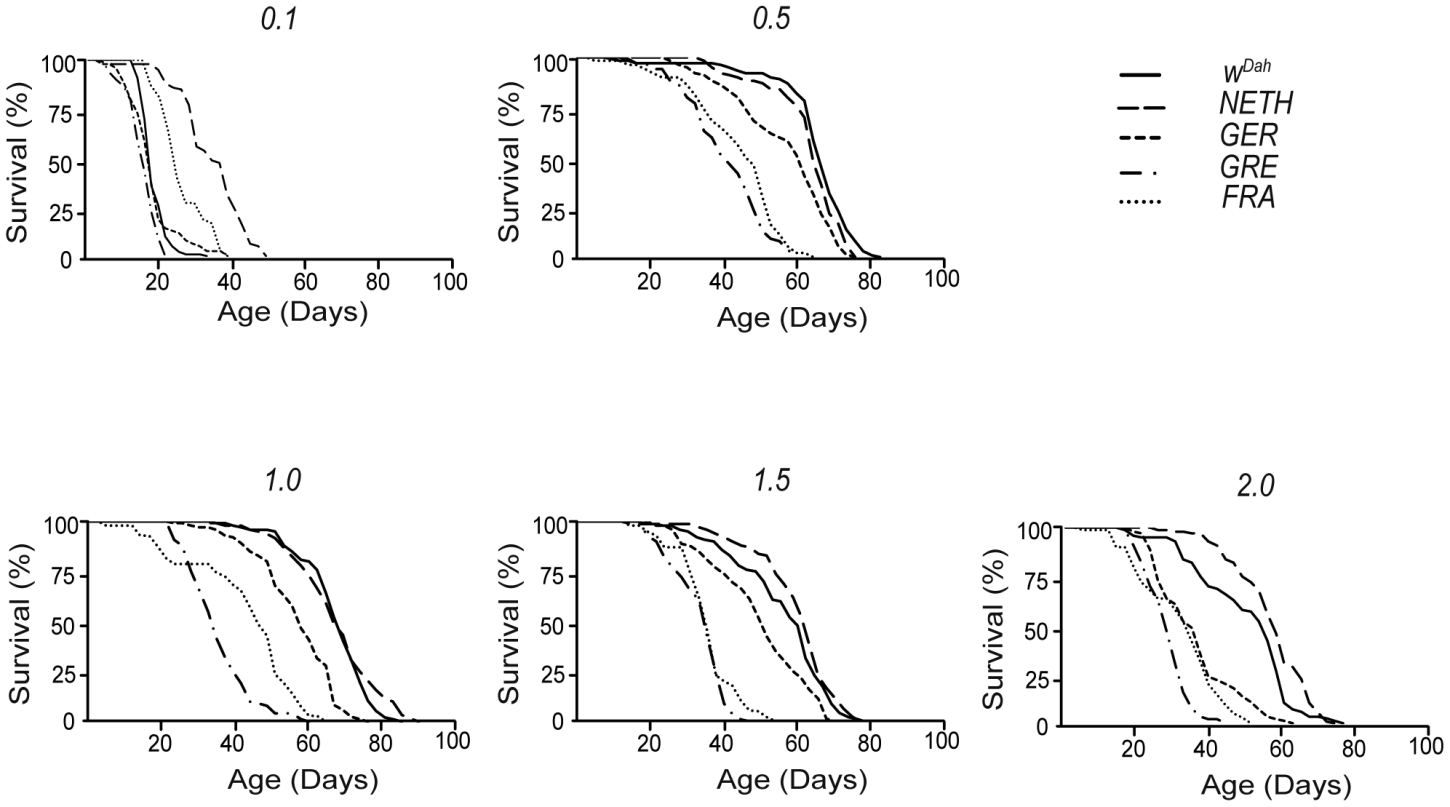
Survival curves for all strains on all foods. *W^Dah^* and *NETH* strains lived longer than any other strain under non-starvation conditions (*p*<10^−4^, log rank test). At 0.1 and 2 yeast concentrations *NETH* strain lived longer than *W^Dah^* (*p*<10^−4^, log rank test), however at other yeast concentrations their lifespans did not differ significantly (*p*>0.05, log rank test).

All four wild-derived strains and the laboratory Dahomey strain showed a typical tent-shaped response of mean lifespan to DR (**Figure 1** **and** **2** **,** for median and maximum lifespan values: **Table 2**). Lifespans peaked at 0.5 or 1.0 yeast concentration and were lowest under starvation (0.1 yeast) and next lowest under the highest yeast concentration (2.0 yeast). Both yeast concentration and geographic origin of the flies affected lifespan [main effect of food on mean lifespan: F = 316.24, DFn = 4, DFd = 225, *p*<1×10^−4^, on maximum lifespan (the mean lifespan of the longest-lived 10% of flies): F = 348.98, DFn = 4, DFd = 225, *p*<1×10^−4^. Main effect of strain on mean lifespan: F = 236.9, DFn = 4, DFd = 225, *p*<1×10^−4^, on maximum lifespan: F = 228.52, DFn = 4, DFd = 225, *p*<1×10^−4^, two-way ANOVA]. Irrespective of yeast concentration, *NETH* and *W^Dah^* had similar mean lifespan values (linear contrasts with Bonferroni's post test, *p* = 0.189) and higher than any other strain compared (linear contrasts with Bonferroni's post test, *p*<0.05). However, at yeast concentrations 0.1 and 2.0 *NETH* strain was significantly longer-lived than strain *W^Dah^* (**Figure 2**). Strain *GER* was longer-lived than strain *CRE* (linear contrasts with Bonferroni's post test, *p*<0.001) but not compared to strain *FRA* (linear contrasts with Bonferroni's post test, *p* = 0.059). Lifespans of strains *FRA* and *GRE* did not differ significantly (linear contrasts with Bonferroni's post test, *p* = 0.286). Yeast concentration 0.1 was the food level at which mean lifespans had the lowest values (linear contrasts with Bonferroni's post test, *p* = 0.001), followed by yeast concentration 2.0 (linear contrasts with Bonferroni's post test, *p* = 0.001, compared to yeast concentration 1.5: *p* = 0.035). At yeast concentrations 0.5 and 1.0 lifespans were longer than under any other food level (linear contrasts with Bonferroni's post test, *p*<0.05), but they did not differ between them (linear contrasts with Bonferroni's post test, *p* = 1).

**Table 2:** Effect of DR on median and maximum lifespan of *Drosophila melanogaster* wild-derived strains.

			Yeast concentration							
	0.1		0.5		1.0		1.5		2.0	
	Median lifespan (days)	Maximum lifespan (days)	Median lifespan (days)	Maximum lifespan (days)	Median lifespan (days)	Maximum lifespan (days)	Median lifespan (days)	Maximum lifespan (days)	Median lifespan (days)	Maximum lifespan (days)
**w^Dah^**	21	28	67	78	69	79	60	70	55	66
**FRA**	25	36	50	62	50	62	36	52	36	50
**GER**	20	37	62	72	58	67	51	67	34	55
**GRE**	19	23	42	60	35	51	35	39	29	34
**NETH**	36	48	64	74	67	85	62	74	57	70

A yeast concentration range of 0.1 to 2.0 captured the DR response of all strains. Both median and maximum lifespans were affected by yeast concentration, in all cases. Maximum lifespan for *W^Dah^*: F = 114.5, R^2^ = 0.9106, *p*<1×10^−4^, for *FRA*: F = 52.76, R^2^ = 0.8242, *p*<1×10^−4^, for *GRE*: F = 58.22, R^2^ = 0.8381, *p*<1×10^−4^, for *GER*: F = 91.41, R^2^ = 0.8904, *p*<1×10^−4^, for *NETH*: F = 48.92, R^2^ = 0.8130, *p*<1×10^−4^, one-way ANOVA test, *n* = 100). Maximum lifespan was calculated as the average lifespan of the most long-lived 10% of flies.

**Figure 3:**
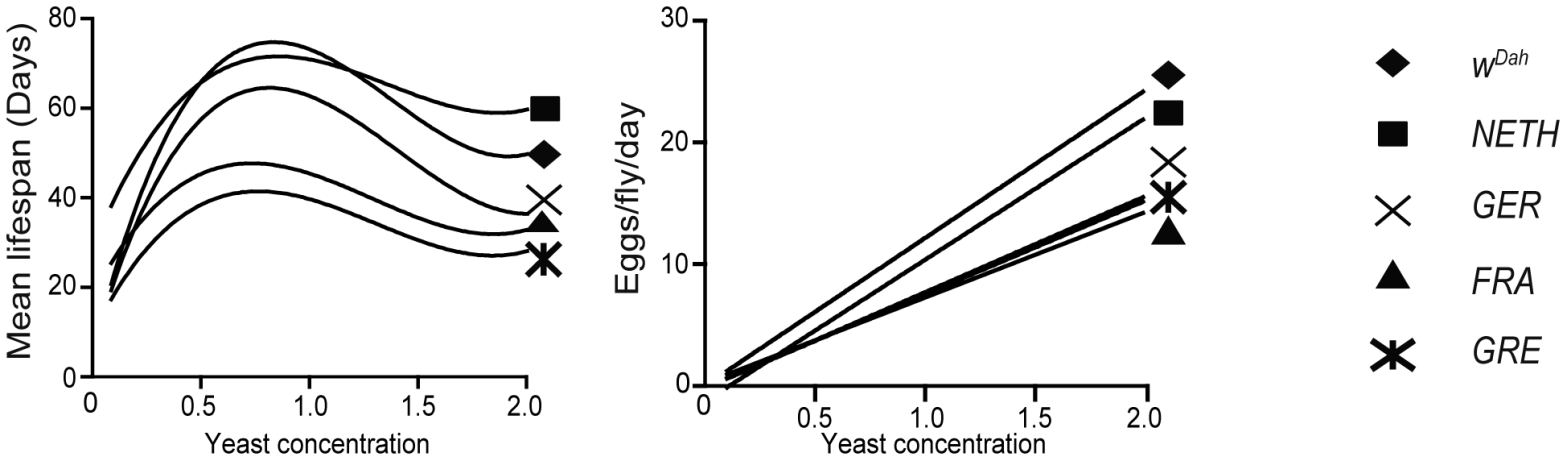
Norms of reaction of wild-derived *Drosophila* populations. Lifespan and fecundity showed different shapes of response to yeast concentration. The effect of yeast concentration on lifespan best fitted a third-order polynomial equation (non-linear regression) and on fecundity a linear equation (linear regression). Goodness of fit is high in both models, as represented by R^2^ values: For mean lifespans: 0.88, 0.74, 0.80, 0.88, 0.79 for *W^Dah^*, *FRA*, *GRE*, *GER*, *NETH* strains respectively. For fecundity: 0.90, 0.87, 0.87, 0.89, 0.91 for *W^Dah^*, *FRA*, *GRE*, *GER*, *NETH* strains respectively. For mean lifespans, best-fit values ranged for B0: 6.6 to 28, for B1: 86.5 to 176, for B2: -81.6 to -151, for B3: 20.9 to 36.8. Linear regression analysis for fecundity: for *W^Dah^*: F = 484.2, DFn = 1, DFd = 48, *p*<1×10^−4^, for *FRA*: F = 335.7, DFn = 1, DFd = 48, *p*<1×10^−4^, for *GRE*: F = 309.9, DFn = 1, DFd = 48, *p*<1×10^−4^, for *GER*: F = 373.6, DFn = 1, DFd = 48, *p*<1×10^−4^, for *NETH*: F = 508.2, DFn = 1, DFd = 48, *p*<1×10^−4^. Lifespan curves and fecundity slopes differed significantly among strains (for lifespan curves: F = 63.23, DFn = 16, DFd = 230, *p*<1×10^−4^, for fecundity slopes: F = 27.10, DFn = 4, DFd = 240, *p*<1×10^−4^).

Food affected lifespan in a strain–dependent way (food vs strain interaction for mean lifespan: F = 10.68, DFn = 16, DFd = 225, *p*<1×10^−4^, for maximum lifespan: F = 11.25, DFn = 16, DFd = 225, *p*<1×10^−4^_,_ two-way ANOVA). Modeling of the DR responses of mean lifespans with polynomial regression analysis showed that the values best fitted a third-order polynomial (cubic) model, described by the equation: Y = B0 + B1*X + B2*X∧ 2 + B3*X∧ 3 (**Figure 3**). Interestingly, mean lifespans of the wild-derived strains peaked at different yeast concentrations (0.5 yeast for *GRE* and *FRA*, at 1.0 yeast for *NETH* and *W^Dah^*, at 0.5 and 1.0 yeast for *GER*) (**Figure 1**) and strains differed at the range of yeast that caused lifespan to decrease and fecundity to increase (DR range, **Table S1**). In conclusion, all strains responded to DR, lifespan values peaked at intermediate yeast concentrations, lifespans differed significantly among strains and the response to yeast concentration varied among strains.

Similarly to what we found with lifespan, both yeast concentration and origin of flies affected fecundity (main effect of food on fecundity: F = 586.82, DFn = 4, DFd = 225, *p*<1×10^−4^, main effect of strain on fecundity: F = 56.83, DFn = 4, DFd = 225, *p*<1×10^−4^). Moreover, food affected fecundity in a strain–dependent way (food X strain interaction: F = 9.11, DFn = 16, DFd = 225, *p*<1×10^−4^_,_ two-way ANOVA). Fecundity values best fitted a straight-line model with *W^Dah^* and *NETH* strains having the steepest slopes (**Figure 3**).

**Figure 4:**
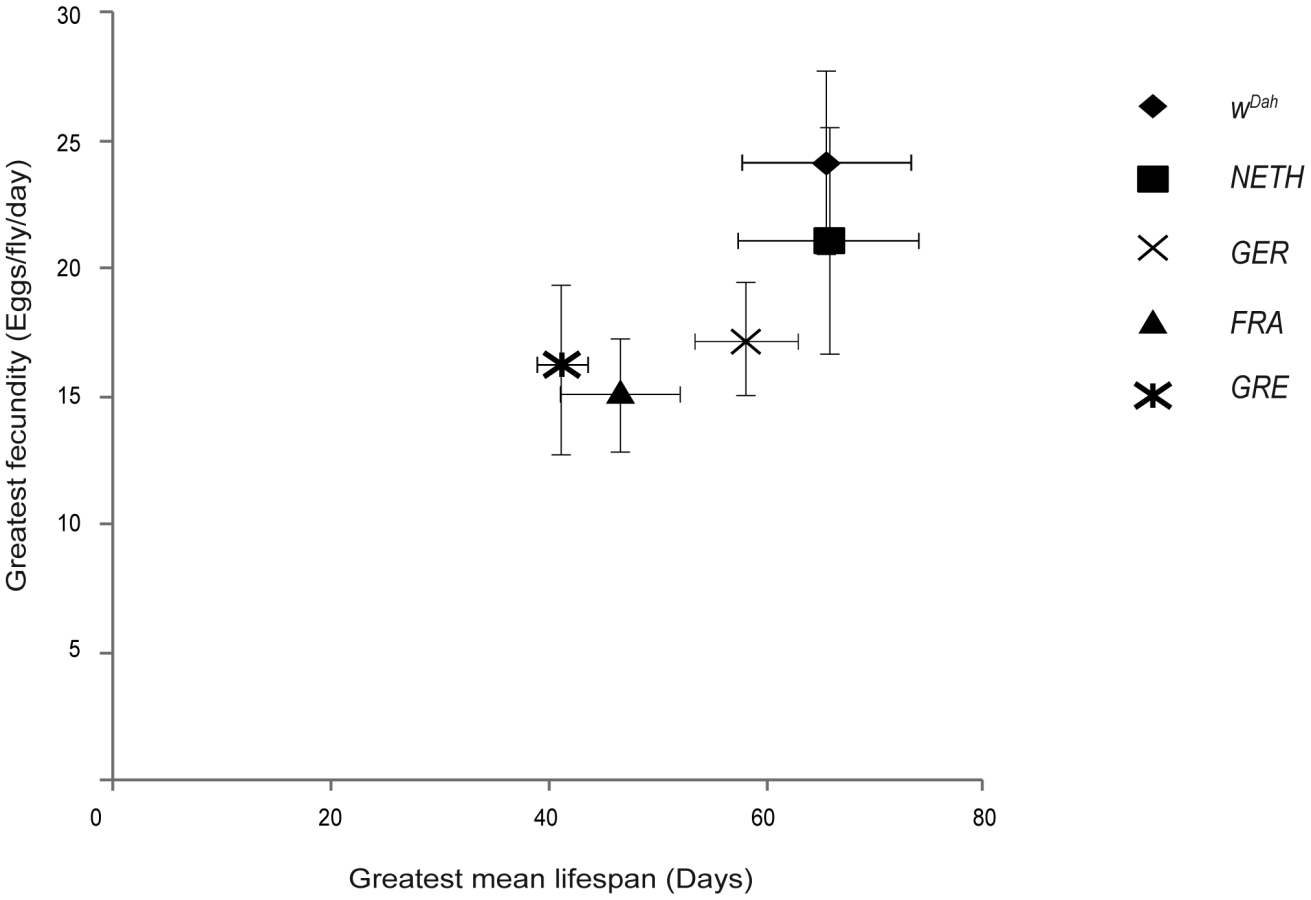
Wild-derived strain *NETH* had similar greatest mean lifespan/fecundity values to laboratory strain *W^Dah^.* Under all food conditions tested in this study, the combined greatest mean lifespan and greatest mean fecundity values were seen in the laboratory-adapted *W^Dah^* and wild-derived *NETH* strains, compared to each of the other strains (for lifespan: *p*<0.001, log rank test, for fecundity: *p*<0.01, one-way ANOVA with Bonferroni's Multiple Comparison test). Greatest mean lifespans for *GRE*, *GER* and *FRA* strains were obtained from 0.5 yeast concentration food, while for *W^Dah^* and *NETH* strains from 1.0 yeast concentration. For all strains greatest fecundity occurred at 2.0 yeast concentration food. Wild-derived strain *NETH* had similar greatest mean lifespan/mean fecundity values to the laboratory strain *W^Dah^* (for lifespan: *p*>0.05, log rank test, for fecundity: *p*>0.05, one-way ANOVA with Bonferroni's Multiple Comparison test). Data are derived from results shown in Figures 1 and 2. Error bars indicate standard deviation (SD).

Greatest lifespan (mean lifespan of each strain at yeast concentration at which lifespan peaked) and greatest fecundity (mean fecundity at yeast concentration 2.0) varied significantly among strains (**Figure 4**). *W^Dah^* and *NETH* flies had the combination of the greatest lifespan and greatest fecundity. Thus, although the lifespan and fecundity of wild-derived strains responded to DR, the exact form of the response, and the greatest mean lifespan/reproductive output they achieved, varied.

## Discussion

In this study we tested whether fly strains recently captured in nature responded to DR, or whether the DR responses of laboratory strains are instead an artifact of adaptation to laboratory conditions. All four wild-derived strains tested were reared for only two generations in the laboratory prior to DR, an insufficient time for laboratory adaptation of life history traits to occur [11]. All strains showed a tent-shaped response of lifespan to DR, comparable to that of a laboratory-adapted strain used as a control, and to other laboratory strains previously studied [20]. Thus we conclude that these two DR responses of *Drosophila* are not an artifact of laboratory culture, as has also been previously demonstrated for *C. elegans* [12].

Greatest mean lifespan and fecundity values were higher in the laboratory-adapted *W^Dah^* strain, but also in the wild-derived strain *NETH*. Higher fecundity of the *W^Dah^* strain could be in part explained by the strain’s history of culture on laboratory food medium, since female flies show higher fecundity on the food medium on which they have evolved [24]. However, strain *NETH* showed similar lifespan and fecundity values to *W^Dah^*, under most food conditions and, interestingly, under starvation and highest yeast concentration food these flies were the longest-lived. Thus, wild-derived strains can reach comparable levels of lifespan and fecundity to laboratory strains.

To our knowledge, the responses of wild-derived populations of *Drosophila melanogaster* to DR have not been previously studied. However, findings in DR experiments with wild-derived [13] or largely inbred [14] rodent strains, subjected to a single level of DR, raised the possibility that lifespan-extension by DR may be a consequence of laboratory adaptation and/or inbreeding. In the study of Harper et al [13] proteins and micronutrients were added to the DR diet, which might have reduced the effect of DR. Additionally, the single level of DR used in these experiments may also have masked a normal set of DR responses, given that the food dilution at which lifespan peaks can vary between strains [22]. Strong DR may have caused nutritional deficiencies in some wild-derived strains and inbreeding depression could both lessen the beneficial effect of DR on lifespan and cause to the food intake at which lifespan peaked to vary [14]. Indeed, the fly strains used in our study showed both variation in the yeast concentration at which lifespan peaked and different values of greatest lifespan/fecundity, as has been previously reported for laboratory strains [16]. Thus, genotype and/or life history can affect the interaction between food and lifespan, and a wide range of food dilutions should be tested to capture the DR response of a population.

## Supporting Information

Table S1 **Statistical significance of DR-mediated changes in fecundity and lifespan of** ***Drosophila melanogaster*** **strains.** The yeast concentration from 0.5 to 0.1 is excluded from the DR range estimation. For fecundity a Student’s two-tailed t-test was used. For lifespans a Log Rank test was used. The statistically significant values are shown in italics (*n* = 100, significance level: *p*<0.05).
(DOC)
