# Supplementary material for: Dietary Restriction Extends Lifespan in Wild-Derived Populations of Drosophila melanogaster
Source: PLoS One. 2013 Sep 10;8(9):e74681. doi: 10.1371/journal.pone.0074681 (PMC3769260; doi:10.1371/journal.pone.0074681)
Supplement: Table S1 — Statistical significance of DR-mediated changes in fecundity and lifespan of Drosophila melanogaster strains. The yeast concentration from 0.5 to 0.1 is excluded from the DR range estimation. For fecundity a Student’s two-tailed t-test was used. For lifespans a Log Rank test was used. The statistically significant values are shown in italics (n = 100, significance level: p<0.05). (DOC) [file pone.0074681.s001.doc]

|  |  |  |  |  |  |  |  |  |  |  |  |
| --- | --- | --- | --- | --- | --- | --- | --- | --- | --- | --- | --- |

**Table S1.** Statistical significance of DR-mediated changes in fecundity and lifespan of *Drosophila melanogaster* strains.
